# Accuracy of a score predicting the presence of an atypical pathogen in hospitalized patients with moderately severe community-acquired pneumonia

**DOI:** 10.1186/s12879-022-07423-1

**Published:** 2022-05-03

**Authors:** Aline Chauffard, Pierre-Olivier Bridevaux, Sebastian Carballo, Virginie Prendki, Jean-Luc Reny, Jérôme Stirnemann, Nicolas Garin

**Affiliations:** 1 Faculty of Medicine, University of Geneva, Geneva, Switzerland; 2 Service de Pneumologie, Centre Hospitalier du Valais Romand, Hôpital du Valais, Sion, Switzerland; 3 Division of General Internal Medicine, Hôpitaux Universitaires de Genève, Geneva, Switzerland; 4 Division of Internal Medicine, Hôpital Riviera Chablais, Rennaz, Switzerland; 5 Service de Médecine Interne, Centre Hospitalier de Rennaz, Rte du Vieux Séquoia 20, 1847 Rennaz, Switzerland; 6 Division of Infectious Diseases, Geneva University Hospitals, Geneva, Switzerland

**Keywords:** Community-acquired pneumonia, Atypical pathogen, Legionella pneumophila, Mycoplasma pneumoniae, Predictive factor, Predictive score

## Abstract

**Background:**

Atypical pathogens (AP), present in some patients with community-acquired pneumonia (CAP), are intrinsically resistant to betalactam drugs, the mainstay of empirical antibiotic treatment. Adding antibiotic coverage for AP increases the risk of adverse effects and antimicrobial selection pressure, while withholding such coverage may worsen the prognosis if an AP is causative. A clinical model predicting the presence of AP would allow targeting atypical coverage for patients most likely to benefit.

**Methods:**

This is a secondary analysis of a multicentric randomized controlled trial that included 580 adults patients hospitalized for CAP. A predictive score was built using independent predictive factors for AP identified through multivariate analysis. Accuracy of the score was assessed using area under the receiver operating curve (AUROC), sensitivity, and specificity.

**Results:**

Prevalence of AP was 5.3%. Age < 75 years (OR 2.7, 95% CI 1.2–6.2), heart failure (OR 2.6, 95% CI 1.1–6.1), absence of chest pain (OR 3.0, 95% CI 1.1–8.2), natremia < 135 mmol/L (OR 3.0, 95% CI 1.4–6.6) and contracting the disease in autumn (OR 2.7, 95% CI 1.3–5.9) were independently associated with AP. A predictive score using these factors had an AUROC of 0.78 (95% CI 0.71–0.85). A score of 0 or 1 (present in 33% of patients) had 100% sensitivity and 35% specificity.

**Conclusion:**

Use of a score built on easily obtained clinical and laboratory data would allow safe withholding of atypical antibiotic coverage in a significant number of patients, with an expected positive impact on bacterial resistance and drug adverse effects.

*Trial registration: *NCT00818610.

## Introduction

Beta lactam drugs are the mainstay of empirical antibiotic treatment for community-acquired pneumonia (CAP). Beta lactams provide coverage for *Streptococcus pneumoniae*, the most frequently identified bacterial pathogen, and for other typical pathogens (e.g. *Hemophilus influenzae*; *Moraxella catarrhalis*) [1–3]. However other causative agents named atypical pathogens such as *Mycoplasma pneumoniae, Chlamydophila pneumoniae* and *Legionella pneumophila* are intrinsically resistant to beta lactam drugs [4]. Atypical pathogens can usually be treated with macrolides, fluoroquinolones, or doxycycline. *S.pneumoniae* and other typical pathogens cannot be reliably discriminated from atypical pathogens on clinical presentation. [5] The overall prevalence of CAP caused by an atypical pathogen among adults is 10–20%, with wide variations between studies [4, 6]. The two more frequent atypical pathogens are *L. pneumophila* and *M. pneumoniae*, the prevalence of *C. pneumoniae* being less than 1% in recent studies [7, 8]. While *M. pneumoniae* causes mostly a mild and self-limiting disease*, L. pneumophila* can cause severe CAP [9, 10].

A microbiological diagnosis is usually not available immediately upon patient’s admission and the antimicrobial therapy is therefore begun empirically. Moreover, with more than half of all pneumonia not having a proven microbiological aetiology, the full course of the antibiotic treatment often remains empirical [3, 11].

Though current treatment guidelines recommend empiric coverage of both typical and atypical pathogens for severe CAP, adding empiric coverage for atypical pathogens in moderately severe CAP, i.e. patients hospitalized for pneumonia in a non-critical care setting, is left to physician’s judgement. [12–14] Adding coverage for atypical pathogens implies combination therapy of a beta lactam with a macrolide or doxycycline, or monotherapy with a respiratory fluoroquinolone.

Thus, there is a dilemma between covering atypical pathogens in all patients with moderately severe CAP to avoid the potential severe outcome of untreated *L. pneumophila* (and to a lesser extent *M. pneumoniae*) and to shorten symptom duration, and unnecessarily broadening the antibiotic coverage, thus increasing the risk of adverse effects and promoting bacterial resistance. A simple prediction model aiming to exclude the presence of atypical pathogens based on demographic and clinical information routinely obtained upon emergency room admission would allow targeting atypical coverage towards patients most likely to benefit. While a few scores predicting the presence of *L. pneumophila* have been described, none have been published to our knowledge to predict the presence of *M. pneumoniae* in adults. We aimed to derive a score predicting the presence of *L.pneumophila* or *M.pneumoniae* from a prospective cohort of patients hospitalized for CAP.

## Methods

### Population and tests

We conducted a secondary analysis of a Swiss multicentric randomized controlled trial testing two empiric antimicrobial treatment strategies in adult patients hospitalized for moderately severe CAP from 2009 to 2013 (NCT00818610) [15]. The study was approved by the competent ethics committee and conducted in 6 acute care hospitals, including two university hospitals from 2009 to 2013. Included patients had two or more symptoms or signs of pneumonia and a new lung infiltrate demonstrated on conventional chest radiography. Patients were excluded if they had severe immunosuppression, were hospitalized during the last 14 days, lived in a nursing home, had been treated with any antibiotic in the last 48 h or had severe pneumonia (Pneumonia Severity Index (PSI) category V or direct admission to the intensive care unit).

Upon arrival, two pairs of blood cultures, a urine sample for the detection of antigens for *L. pneumophila*, and an oropharyngeal swab for the detection of *C. pneumoniae* and *M. pneumoniae* by polymerase chain reaction (PCR) were obtained for all patients. Urine samples were also tested for *S.pneumoniae* antigen detection in a majority of patients. Sputum was obtained for culture in all patients able to expectorate, and pleural fluid was sampled for culture according to recommendations. Use of specific culture media for *Legionella sp* or PCR testing of sputum samples was done upon physician request. PCR for respiratory viruses on either sputum or oropharyngeal swab could be performed if requested by the physician in charge of the patient but were not systematic. Demographic data (including the presence of comorbidities), vital signs, the results of routinely obtained blood tests and of chest X-rays were collected prospectively according to the protocol.

### Definitions

An atypical pathogen (AP) was considered the causative agent of pneumonia if the patient had a positive culture or PCR or urinary antigen detection for *L. pneumophila*; or if the oropharyngeal swab was positive for *M. pneumoniae* in the absence of another detected bacterial pathogen. Patients with another aetiology or no identified pathogen formed the non-atypical pathogen group (NAP).

### Statistical analysis

All patients were included in the analysis. We used frequencies, percentage, mean with standard deviations and median with interquartile range for descriptive purposes. Characteristics of AP and NAP patients were compared using Mann–Whitney *U* test for continuous variables, and Fisher's exact test or Chi-square test for categorical variables, as appropriate.

Factors associated with AP with a p-value < 0.1 were entered in a multivariate logistic regression model, using backward elimination. Because the number of AP patients was 31, a maximum of 6 variables were accepted in the final model [16]. The baseline dataset included 580 patients. No imputation was made for missing values (complete case analysis). Goodness-of-Fit was assessed with Hosmer and Lemeshow Test.

A predictive score was elaborated using the independent predictive factors identified in the multivariate analysis. The continuous variables were dichotomized at the best discriminative cut-off selected using the receiver operating characteristic (ROC) curve and Youden Index.

Each predictive factor was assigned a number of points weighted according to Beta coefficient, and the final score was computed by summing the points of all factors present in an individual patient. A ROC curve for the score was built. For each possible cut-off, the sensitivity, specificity, positive and negative predictive value, positive and negative likelihood ratio, and diagnostic odds-ratio were computed [17]. Because the main aim of this score was to exclude the presence of an atypical pathogen, the threshold with a high sensitivity and negative predictive value was sought.

The study sample was determined by the design of the original study. All p-values are two-tailed and considered significant for p < 0.05. Data were analysed using IBM SPSS Statistics for Windows, Version 25.0. Armonk, NY: IBM Corp. Released 2017.

## Results

Five hundred eighty patients were included in the original cohort. Median age was 76 years (IQR 64–84), and median PSI score was 85 (IQR 68–103). An oropharyngeal swab for detection of *M. pneumoniae* or *C. pneumoniae* by PCR was available in 564 patients (97%), and urine was tested for the presence of *L. pneumophila* antigen in 545 (94%). Atypical pathogens were diagnosed in 31 patients: 16 were *L. pneumophila* and 15 M*. pneumoniae*. No patient was diagnosed with C*. pneumoniae*. The NAP group was composed of 549 patients with 149 having a proven aetiology (88 *S. pneumoniae* and 61 other bacteria) and 400 with unknown aetiology after all microbiological investigations, including a negative oropharyngeal swab for *M. pneumoniae* and urinary antigen testing for *L. pneumophila*.

### Patients’ characteristics

Univariate comparisons between AP and NAP are displayed in Table 1. There was a higher temperature (p = 0.03) and a trend towards a younger age in the AP group (p = 0.09). Absence of chest pain (p = 0.05) and presence of acute confusion (p < 0.01) were more frequent in the AP group, and there was a trend towards a higher prevalence of non-productive cough in the AP group.

**Table 1:** Characteristics of patients with atypical (AP) or non-atypical (NAP) pneumonia (frequencies with percentage, and means with standard deviations)

Variable	AP (N = 31)	NAP (N = 549)	P-value
Age (years)	67 (17)	72 (16)	0.09
Gender (male)	19 (61)	314 (57)	0.65
Heart failure	11 (36)	105 (19)	**0.03**
Chronic obstructive pulmonary disease	5 (16)	117 (21)	0.49
Chronic liver disease	1 (3)	6 (1)	0.29
Active cancer	3 (10)	35 (6)	0.47
Chronic renal disease	5 (16)	83 (15)	0.88
Diabetes	3 (10)	93 (17)	0.29
Neurological disease	5 (16)	61 (11)	0.39
Alcohol abuse	5 (16)	54 (10)	0.26
Number of comorbidities			
0	12 (39)	217 (40)	
1	8 (26)	177 (32)	0.63
> 1	11 (36)	155 (28)	
Previous antibiotic treatment	0	26 (5)	0.22
Cough	24 (77)	457 (84)	0.38
Sputum	15 (48)	326 (60)	0.22
Chest pain	5 (17)	187 (34)	**0.05**
Dyspnea	22 (73)	383 (70)	0.70
Fever	24 (77)	370 (67)	0.25
Confusion	5 (16)	17 (3)	** < 0.01**
Temperature	38.2 (1.3)	37.9 (1.0)	**0.03**
Heart rate	99 (20)	98 (20)	0.85
Respiratory rate	25 (6)	24 (6)	0.64
Systolic blood pressure	135 (17)	133 (24)	0.64
Diastolic blood pressure	77 (11)	73 (14)	0.11
Hypoxemia (SpO2 < 90% on room air)	12 (40)	279 (52)	0.21
Focal signs on chest examination	24 (77)	461 (84)	0.33
Natremia (mmol/L) (N = 579)	133.4 (4)	135.9 (4)	** < 0.01**
Urea (mmol/L) (N = 569)	7.3 (4)	7.6 (5)	0.52
Glucose (mmol/L) (N = 565)	7.6 (2)	7.6 (3)	0.59
Leukocytes count (G/L)	11.5 (4.3)	13.6 (6.4)	0.09
Hematocrit (%)	39.3 (5)	39.2 (5)	0.88
Platelets count (G/L)	212.2 (89)	233.8 (97)	0.12
Procalcitonin (ug/L) (N = 540)	1.5 (3)	3.8 (14)	0.81
C-reactive protein (mg/ L) (N = 230)	265.6 (112)	180.4 (138)	** < 0.01**
Pleural effusion	6 (19)	91 (17)	0.69
Pneumonia Severity Index score	86.3 (24)	84.2 (25)	0.59
Season of inclusion			
Winter	5 (16)	207 (38)	**0.03**
Spring	6 (19)	123 (22)	
Summer	5 (16)	76 (14)	
Autumn	15 (48)	143 (26)	

The p-values in bold letters are considered as significant and the ones underlined are inferior to 0.1 and are therefore also used for the multivariate analysis

All data were available for all patients except when stated otherwise

A lower natremia was significantly associated with AP (p < 0.01). CRP was significantly higher in the AP group (p < 0.01). The leukocytes count tended to be lower in the AP group (p = 0.09).

Other biomarkers such as urea, glucose, hematocrit, procalcitonin and platelet count, or presence of a pleural effusion on chest X-rays, did not significantly differ between groups.

Heart failure was significantly more prevalent in the AP group (p = 0.03). All other comorbidities such as chronic obstructive pulmonary disease (COPD), chronic liver disease, chronic renal disease and active cancer did not significantly differ between the aetiological groups, nor did the number of individual comorbidities, alcohol abuse, or previous antibiotic use.

When looking at the seasonal distribution of the different aetiologies, the atypical pathogens were more prevalent in autumn (p < 0.01).

### Predictive model specification

Nine variables differed between the AP and NAP groups with a p < 0.1. We excluded CRP because it was only available in 230 patients, and confusion, because less than 10% of all patients presented this symptom. Therefore, 7 variables were entered in the multivariate model: age, heart failure, chest pain, temperature, inclusion in autumn, natremia and leucocytes count. Temperature and leucocytes count were dismissed by the backward selection, leaving 5 variables in the final model with a confirmed goodness-of-fit (p = 0.30 by Hosmer and Lemeshow Test) (Table 2). Two patients were excluded by the procedure due to an incomplete data set.

**Table 2:** Multivariate association of variables for AP vs. NAP

Variable	Odds ratio	95% CI	Beta coefficient	P-value
Age < 75 years	2.674	1.159–6.171	0.984	0.021
Heart failure	2.567	1.089–6.054	0.943	0.031
Absence of chest pain	3.001	1.099–8.195	1.099	0.032
Autumn	2.708	1.250–5.867	0.996	0.012
Sodium < 135 mmol/L	2.979	1.351–6.568	1.092	0.007

Both the presence of heart failure (p = 0.02) and contracting pneumonia during autumnal months (p = 0.01) were positive predictive factors for the presence of atypical pathogens. The presence of chest pain (p = 0.01), advanced age (p < 0.01) and higher natremia (p = 0.01) were independent negative predictive factors.

Using the Youden Index, the optimal cut-off were 75 years for age and 135 mmol/L for natremia. After dichotomization of the two variables, all variables remained independent predictive factors in multivariate analysis.

Because the five independent predictive factors can be easily obtained at admission, they were incorporated in a predictive score. Beta coefficients being all close to one, the same weight was allocated to all factors, and the score equals the number of factors present, for a total ranging between zero and five. The mnemonic “CASH-75” can be used to recall the factors composing the score (Table 3). The higher the score, the more probable the presence of an atypical bacteria (Table 4).

**Table 3:** The CASH-75 predictive score

CASH-75	Clinical feature	Weight
C	Absence of **C**hest pain	1
A	Contracting the disease in **A**utumn	1
S	**S**odium < 135 mmol/L	1
H	**H**eart failure	1
75	Age < **75** years	1

**Table 4:** Prevalence of an atypical aetiology according to CASH-75 score

Score	Number of patients (Frequencies (%))		Total
	NAP group	AP group	
0	23 (100)	0	23
1	170 (100)	0	170
2	207 (95.8)	9 (4.2)	216
3	118 (89.4)	14 (10.6)	132
4	27 (79.4)	7 (20.6)	34
5	3 (100)	0	3
Total	548	30 (5.2)	578

**Fig. 1:**
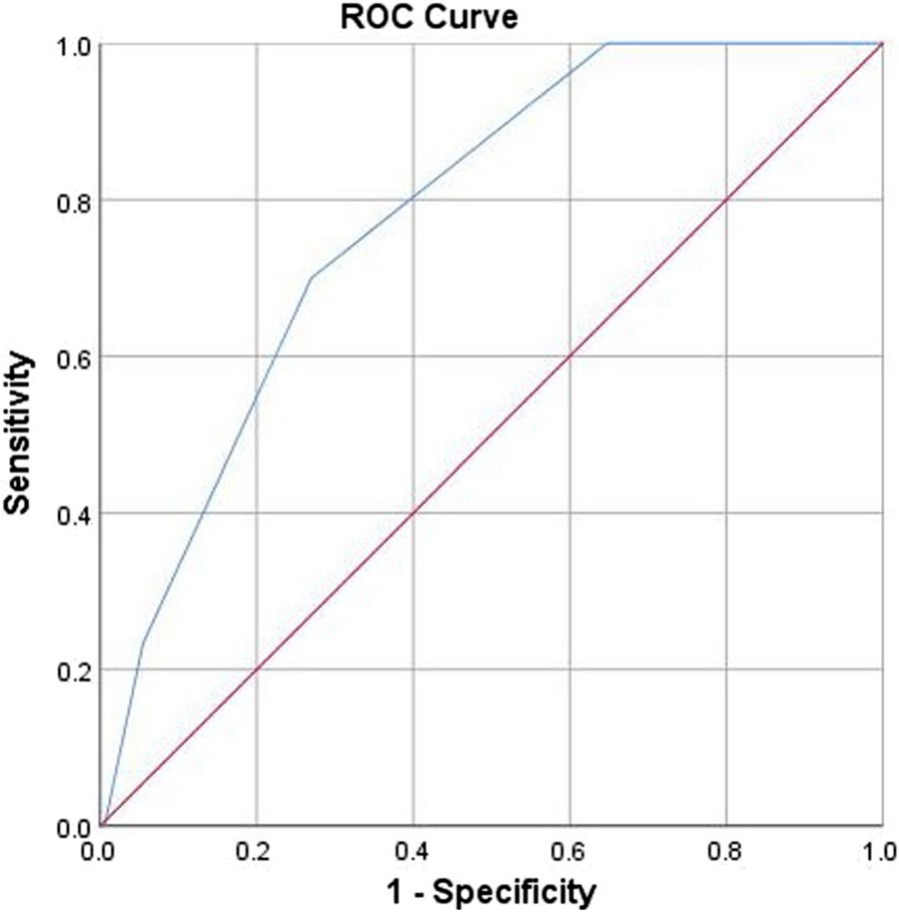
ROC curve of the CASH-75 score

The performance characteristics of the score were then calculated for each cut-off (Table 5). The ROC curve of the score had an area under the curve (AUC) of 0.78 (95% CI = 0.71–0.85) (Fig. 1).

**Table 5:** Performances of the CASH-75 score according to different cut-offs

Cut-off	Sensitivity (%)	Specificity (%)	PPV (%)	NPV (%)	LR +	LR-	DOR
< 1	100	4	5	100	1.04	0	+ ∞
< 2	100	35	8	100	1.54	0.35	4.4
< 3	70	73	12	98	2.59	0.41	6.3
< 4	23	94	19	96	3.83	0.82	4.7
< 5	0	99	0	95	0	1.01	0

*PPV* positive predictive value, *NPV* negative predictive value, *LR +* positive likelihood ratio, *LR−* negative likelihood ratio, *DOR* diagnostic odds-ratio

With a cut-off set at < 2, a negative test would predict the absence of an atypical pathogen with a sensitivity of 100% and a negative predictive value of 100%. With a cut-off of < 3, the specificity increases to 73%, the sensitivity decreases to 70%, but the negative predictive value remains high at 98%. The cut-off of < 3 has the highest diagnostic odds-ratio.

## Discussion

We found that heart failure, absence of chest pain, contracting the disease in autumn, lower natremia and younger age were all independently associated with an atypical aetiology of pneumonia in hospitalized, adult, immunocompetent patients with moderately severe CAP.

A younger age, a higher body temperature and a lower leukocytes count have been associated with the presence of an atypical pathogen in previous studies [18]. Hyponatremia [19–26], absence of chest pain [23, 24, 27, 28], and contracting the disease in summer or autumn [29–31] are well-described predicting factors for *Legionella sp* infection.

Chronic heart failure as a risk factor for AP was an unexpected finding. Heart failure has been associated with a significantly lower risk of *Legionella sp* infection in some, [20, 25] but not all studies. [32, 33] The relations between pneumonia and heart failure are complex and multidirectional. Heart failure is a known risk factor for pneumonia, and pneumonia frequently triggers acute heart failure and other cardiovascular events. Whether the observed association between chronic heart failure and increased risk of AP is causal or a chance finding should be investigated in other populations.

Confusion was significantly associated with the presence of an atypical pathogen in univariate, as extensively described in other studies [22, 34, 35]. We chose to exclude confusion form the multivariate analysis because its prevalence was lower in our population than in observational studies, probably because our patients were included in an interventional trial and had to sign an informed consent form. This low incidence meant that the impact of confusion on the multivariate model was expected to be low.

With an AUC of 0.78 (95% CI = 0.71–0.85), the accuracy of the CASH-75 score is comparable with other scores used for predicting the aetiology of pneumonia. The Legionella score proposed by Fiumefreddo et al.[20] has an AUC of 0.86 in the derivation study and of 0.73 and 0.91 in validation studies [25, 36]. Unfortunately, we could not attempt to validate this score in our population, because LDH and C-reactive protein (two of its 6 criteria) were not measured in all patients. The Winthrop-University Hospital (WUH) criteria by Cunha[37] predicts the presence of *L. pneumophila* with 21 different clinical features and a weighted point system. While the original study does not state accuracy, a validation study has described an AUC between 0.68 and 0.72 [28]. The CBPIS scoring system is a weighted point system described by Keller et al. with an AUC calculated at 0.76 [22]. The New Score proposed by Saraya et al. was derived on a cohort of only 102 patients, with an AUC between 0.62 and 0.68 [21]. We did not find any score or clinical rule predicting the presence of any atypical pathogens, i.e. not only *L. pneumophila* but also *M. pneumoniae* or *C. pneumoniae*.

We aimed to build a score easy to use at the bedside. All the information needed to compute the CASH-75 score can be obtained through readily available information and routine laboratory tests. The absence of weighting of the different items confers simplicity to this score, enhancing its potential usefulness in a busy clinical setting.

We chose to compare patients with AP to both patients with another aetiology (eg. typical bacterial pathogens) and patients without any identified pathogen. It can be argued that some patients without identified pathogens had viral pneumonia and as such would not need any antibiotic treatment. Though this assumption is probably true, some of these patients may as well have infection with an undetected bacterial pathogen [38, 39]. At present, international guidelines do not recommend withholding antibiotics in patients with viral pneumonia, as associated bacterial infection cannot be reliably ruled-out [12]. As our main aim was to allow safe withholding of atypical coverage in all patients presenting with pneumonia, we thought that it was more appropriate to include in the analysis patients without detected pathogens.

The main usefulness of the CASH-75 score is to exclude an atypical bacteria as the causative pathogen in order to safely withdraw atypical coverage from the empiric antibiotic treatment. For this purpose, there are two candidate cut-offs. The first one, < 2, has a sensitivity and negative predictive value of 100%. Using this cut-off, antibiotic coverage for atypical pathogens would have been avoided in 193/578 (33%) patients with a score of 0 or 1, without missing any cases of atypical pathogen. Based on the highest diagnostic odds-ratio, the ideal cut-off is < 3. Using this cut-off, atypical antibiotic coverage would be withheld in 400/ 578 (69%) patients, at the cost of 9 CAP caused by atypical pathogens not being covered by an adequate antibiotic. Both cut-offs may help reduce the prescription of atypical coverage before obtaining the results of any microbiological investigation. This may lead to less bacterial resistance, less drug adverse effects and drug interactions.

This study has several strengths; it was conducted using a prospective multicentric cohort with thorough adjudication of the presence of pneumonia. The protocol mandated search for typical and atypical pathogens in all patients, hence minimizing the risks of misclassification. Patients were representative of older individuals admitted at the hospital in Switzerland.

Nevertheless, some limitations must be recognized. Not all variables of interest described in the literature were available in our patients. For example, the smoker status remained unknown and the LDH were not measured, both being described in other studies as significant predictive factors for the presence of *L. pneumophila*. Because no systematic attempt was made to detect *L.pneumophila* or *M.pneumoniae* by culture or PCR in the sputum, we cannot completely exclude the presence of these pathogens in the NAP group creating a possible classification bias. However, all patients were tested for the presence of either pathogens with PCR on an oropharyngeal swab (*M. pneumoniae*) or urinary antigen detection (*L. pneumophila*), making significant misclassification unlikely. The diagnosis was confirmed by chest-X ray, which has lower accuracy than CT-scan for pneumonia [40]. However, chest X-ray remains a frequently used tool to confirm pneumonia in clinical studies.

Finally, despite the model fulfilling the goodness-of-fit hypothesis, the total number of patients in the AP group was low, and overfitting of the score is a possibility, reinforcing the need for external validation.

## Conclusion

Some characteristics can help predict an atypical aetiology in patients hospitalized for moderately severe pneumonia. Provided its accuracy is confirmed in other cohorts, the CASH-75 score could help exclude the presence of atypical bacteria using simple, easy to obtain variables. This would enable a safe antibiotic stewardship guided by the CASH-75 score, with beneficial effects on bacterial resistance, drug adverse events and interactions, while maintaining lower costs.

## Data Availability

The datasets generated during the original study and analyzed during the current study are not publicly available due to the absence of adequate repository when the original study was conducted, but are available from the corresponding author on reasonable request.
